# Mitochondrial respiration in peripheral blood mononuclear cells of healthy adult population

**DOI:** 10.1371/journal.pone.0336939

**Published:** 2026-01-05

**Authors:** John H. Lee, Jacob Vine, Lakshman Balaji, Natia Peradze, Nivedha Antony, Yanbo Wang, Andrea Morton, Ari Moskowitz, Michael W. Donnino, Xiaowen Liu

**Affiliations:** 1 Center for Resuscitation Science, Beth Israel Deaconess Medical Center, Boston, Massachusetts, United States of America; 2 Department of Emergency Medicine, Beth Israel Deaconess Medical Center, Boston, Massachusetts, United States of America; 3 Department of Medicine, Division of Critical Care Medicine, Montefiore Medical Center, The Bronx, New York, United States of America; 4 Bronx Center for Critical Care Outcomes and Resuscitation Research, Montefiore Medical Center, The Bronx, New York, United States of America; 5 Department of Medicine, Division of Pulmonary, Critical Care, and Sleep Medicine, Beth Israel Deaconess Medical Center, Boston, Massachusetts, United States of America; Universitat Ramon Llull, SPAIN

## Abstract

Given the potential for mitochondrial medicine as a therapy in various illnesses, mitochondrial tests derived from blood samples have gained increasing value. The aim of this study was to perform an in-depth investigation of mitochondrial respiration in peripheral blood mononuclear cells (PBMCs) of healthy adult participants to characterize mitochondrial respiration in health. Adult participants without acute illness were recruited. PBMCs were isolated and quantitative, real-time measurements of mitochondrial oxygen consumption rate were performed using the Seahorse XF Cell Mito Stress Test Kit with Seahorse XFe96 Analyzer. The study included 184 participants without previously diagnosed medical conditions. There was no association between mitochondrial respiration and sex and age groups (≤ 30 years vs. > 30 years). Maximal and Spare respirations were associated with body mass index (BMI). The findings of this study contribute to the growing body of knowledge on mitochondrial bioenergetics in healthy adults and provide further insights into its association with demographic and anthropometric factors.

## Introduction

Mitochondria are the primary sites of oxygen consumption within cells due to their role in aerobic respiration. Increasing evidence suggests that mitochondria play a central role in various physiologic and pathologic processes in the human body such as regulation of metabolism [1], aging [2], airway diseases [3–5], cardiovascular [6], inflammatory [7], neurodegenerative [8], muscular diseases [9–11], cancers [12], and others. Due to the importance of mitochondria for cellular bioenergetics and overall performance, decrease in mitochondrial function has also been linked to decreased life span [13,14].

One of the most important roles of mitochondria is the regulation of cellular bioenergetics, more specifically, the production of adenosine triphosphate (ATP) by oxidative phosphorylation [15]. Mitochondria produces ATP by metabolizing glucose, carbohydrate and fatty acid via the Krebs cycle to meet cellular energy demands [16–18]. This process is orchestrated by the multi-subunit protein complexes I – IV within the mitochondrial respiratory chain, which transfer protons from the mitochondrial matrix to the intermembrane space to generate mitochondrial membrane potential, which is later utilized by complex V (ATP synthase) to synthesize ATP [19]. In addition to regulation of cellular bioenergetics, mitochondria also play an important role in cellular stress responses that maintain key biological processes in the cell [1]. Therefore, defects in mitochondria due to extreme cellular damage could lay the groundwork for pathogenesis of a multitude of diseases. For example, previous studies have examined the inflammatory process in the setting of obesity which leads to oxidative stress, and in turn results in mitochondrial dysfunction [20–22].

Due to the important role that mitochondria play in critical illnesses and the potential for mitochondrial targeted therapies as a treatment strategy in these illnesses [23–28], there has been increasing interest in real-time assessment of mitochondrial functions derived from blood samples. Peripheral Blood Mononuclear Cells (PBMCs), which include lymphocytes (e.g., T cells, B cells, and NK cells) and monocytes play a crucial role in the immune system. The function and status of PBMCs can be indicative of overall health and provide insights into the nutritional and metabolic status of an individual. PBMCs are widely used in immunological research because they can be easily isolated from blood samples and have the potential to be an ideal cell type for assessing mitochondrial function in the context of critical illness [29]. Several studies have investigated mitochondrial respiration in PBMCs in illness including neurodegenerative [30–32], sepsis [33], type I diabetes [34], end-stage renal disease [35], liver disease [36], and mitochondrial disease [37,38], which showed changes in respiration associated with illnesses. Although several prior studies have included healthy individuals as control groups or as the primary population of interest [33,39–41], their findings have been limited by relatively small sample sizes. Therefore, the goal of this study was to assess the mitochondrial function of a large cohort of healthy individuals by measuring the oxygen consumption rate (OCR) parameters in PBMCs to 1) contribute to the growing body of knowledge on mitochondrial function in healthy adults and 2) to investigate its association with demographic and anthropometric factors, including age, sex, and body mass index (BMI).

## Materials and methods

### Study population

Healthy adult participants (≥ 18 years) without any previously diagnosed medical conditions were recruited for this study. Of note, the participants were recruited as part of three clinical studies conducted at Beth Israel Deaconess Medical Center (BIDMC). Of these three studies, one was a clinical trial and was registered at www.clinicaltrials.gov. The studies were: Study 1 – Corticosteroid therapy in refractory shock following cardiac arrest (NCT00676585), Committee on Clinical Investigation (CCI) Protocol #: 2007P-000227 (study period: 9/17/2007 – current, participants included in this study were recruited from 4/13/2015–3/19/2017), Study 2 – Blood, plasma, and serum repository for use in control comparisons in current and future research studies, CCI Protocol # 2019P-000717 (study period: 10/7/2019 – current, participants included in this study were recruited from 1/20/2020–6/24/2024), and Study 3 – Mitochondrial metabolism in metformin and other pharmacological overdoses, CCI Protocol #: 2015P-000326 (study period: 12/14/2015 – current, participants included in this study were recruited from 2/8/2016–12/3/2020). Written informed consent was obtained from all participants. Baseline demographic and anthropometric data was collected by trained research personnel and recorded into a Research Electronic Data Capture (REDCap) database (REDCap version 4.3.5, Research Electronic Data Capture Consortium, Nashville, TN). Study protocols were approved by the Institutional Review Board (IRB) of BIDMC. Studies were conducted in accordance with the Declaration of Helsinki and the International Conference on Harmonization for Good Clinical Practice.

### Cellular metabolism measurements

Fresh whole blood sample of the study population was processed to isolate PBMCs using the density gradient separation method (Ficoll-Paque premium, GE Healthcare Bio-Science Corp) and Roswell Park Memorial Institute (RPMI) buffer. Real-time quantitative assessment of mitochondrial function and metabolic dysfunction was performed using Seahorse XF Cell Mito Stress Test Kit with Seahorse XFe96 Analyzer (Agilent, Santa Clara, CA, USA) [42,43], which measures the OCR and extracellular acidification rate (ECAR). Fresh whole blood samples were collected and processed to isolate PBMCs using the density gradient separation method (Ficoll-Paque premium, GE Healthcare Bio-Science Corp) and RPMI buffer. After isolation of PBMCs, the cell numbers were counted using Millipore Scepter Handheld Automated Cell Counter and 400,000 cells/well were seeded in a 96-well assay plate pre-coated with CellTak (BD Biosciences) to ensure attachment of the cells to the well. The final volume of medium was brought to 180 µL/well with XF Assay medium supplemented with 5.5mM glucose, 1mM pyruvate, and 4mM L-glutamine.

According to the manufacturer’s instructions, modulators of respiration that target specific components of the electron transport chain (ETC) were serially injected to reveal key parameters of metabolic profile. The interval from 0 min to 21 min measured the basal respiratory rate. At 21 min oligomycin (1 µM), an inhibitor of complex V (ATP synthase) was added. Subsequent decrease in OCR correlates with the mitochondrial respiration associated with cellular ATP production. At 48 min, Carbonyl cyanide-4 (trifluoromethoxy) phenylhydrazone (FCCP, 1 µM), an uncoupler of oxidative phosphorylation in the mitochondria, was added to measure the maximal respiration. FCCP collapses the proton gradient and disrupts the mitochondrial membrane potential. As a result, electron flow through the ETC is uninhibited and oxygen is maximally consumed by complex IV. The FCCP-stimulated OCR was then used to calculate spare respiration, defined as the difference between maximal respiration and basal respiration. Spare respiration is a measure of the cell’s ability to respond to increased energy demand. At 74 min, a mix of rotenone (0.5 µM), a complex I inhibitor, and antimycin A (0.5 µM), a complex Ill inhibitor, were added to inhibit electron transfer thereby completely inhibiting mitochondrial respiration. This allowed us to assess non-mitochondrial respiration. The final values for basal respiration, maximal respiration, and proton leak were calculated by subtracting the non-mitochondrial respiration [44]. Of note, samples with higher ATP-linked respiration than basal respiration were removed from the analysis given this likely indicates measurement error. The concentration of regents used in the assay were determined based on our preliminary experiments and are consistent with those reported in previous studies. A sample Seahorse XF Cell Mito Stress Test profile showing stable OCR at each phase is shown in S1 Fig. The resulting OCR and ECAR values were normalized to protein concentration measured for each well using a standard bicinchoninic acid-based protein assay in addition to the internal normalization through pre-assay cell count at the time of seeding. The reported OCRs are in pmol/min/µg of protein.

### Statistical analysis

The outcome variables of interest were six mitochondrial respiration variables: basal respiration, maximal respiration, ATP-linked respiration, spare respiratory capacity, proton leak, and non-mitochondrial respiration. The distributions of the outcome variables were examined using Shapiro-Wilk test and histograms, and evidence against the assumption of normality was noted. Therefore, the variables are described using medians and interquartile ranges, and analyzed using non-parametric statistics. We examined the univariate associations between these outcome variables and each of three different predictor variables: 1) sex (categorical variable with two levels: female and male), 2) age (as an ordinal variable with two levels: ≤ 30 years and > 30 years and as a continuous variable), and 3) body mass index (BMI) (as an ordinal variable with two levels: ≤ 30 kg/m^2^ (underweight and healthy) and > 30 kg/m^2^ (overweight – morbidly obese) and as a continuous variable. The associations between the outcome variables and sex, age groups, and BMI groups were assessed using the Wilcoxon Rank-Sum test. We conducted further analyses to account for potential confounding variables using rank-based regression model [45,46] to preserve the sample size. Associations with age were adjusted for sex and BMI, associations with sex were adjusted for age and BMI, and association with BMI were adjusted for age and sex. The association between the outcome variables and age and BMI as continuous variables were assessed using quantile regression. All statistical analysis was performed in R-4.1.1 and p-values < 0.05 were considered statistically significant.

## Results

A total of 184 participants were included in the analysis. The majority were between 18 and 30 years of age (75.0%) and female (78.8%). Most participants identified as White (88.0%) and non-Hispanic (91.3%). Additional baseline demographic and anthropometric parameters of the study participants are presented in Table 1.

**Table 1:** Baseline characteristics.

	Female (n = 145)	Male (n = 39)	Total (n = 184)
Age (median, IQR)	26.0 [24.0, 31.0]	26.0 [24.0, 28.5]	26.0 [24.0, 30.3]
Age Group (n, %)			
18–30 years (n, %)	105 (72.4%)	33 (84.6%)	138 (75.0%)
31–40 years (n, %)	26 (17.9%)	5 (13.8%)	31 (16.8%)
41–50 years (n, %)	7 (4.8%)	0 (0.0%)	7 (3.8%)
51–70 years (n, %)	7 (4.8%)	1 (2.6%)	8 (4.3%)
Race (n, %)			
AI/AN	3 (2.1%)	0 (0.0%)	3 (1.6%)
Asian	0 (0.0%)	2 (5.1%)	2 (1.1%)
Black/African American	7 (4.8%)	3 (7.7%)	10 (5.4%)
White	131 (90.3%)	31 (79.5%)	162 (88.0%)
More than one race	3 (2.1%)	2 (5.1%)	5 (2.7%)
Unknown/not reported	1 (0.7%)	1 (2.6%)	2 (1.1%)
Ethnicity (n, %)			
Hispanic	13 (9.0%)	3 (7.7%)	16 (8.7%)
Non-Hispanic	132 (91.0%)	36 (92.3%)	168 (91.3%)
Anthropometrics data (median, IQR)			
Weight (kg) ^†^	62.4 [57.1, 72.6]	87.3 [78.5, 95.5]	65.8 [59.0, 79.4]
Height (cm) ^‡^	165.0 [160.0, 170.0]	180.2 [178.0, 183.5]	168.0 [163.0, 175.0]
BMI (kg/m^2^) ^§^	22.95 [21.30, 26.51]	25.7 [23.4, 30.0]	23.5 [21.50, 27.06]

^†^Weight information is missing in 10 participants. ^‡^Height information missing in 11 participants. ^§^BMI missing in 13 participants. IQR, interquartile range; AI, American Indian; AN, Alaska Native; BMI, body mass index.

### Mitochondrial respiration and sex, age, and BMI

**Fig 1:**
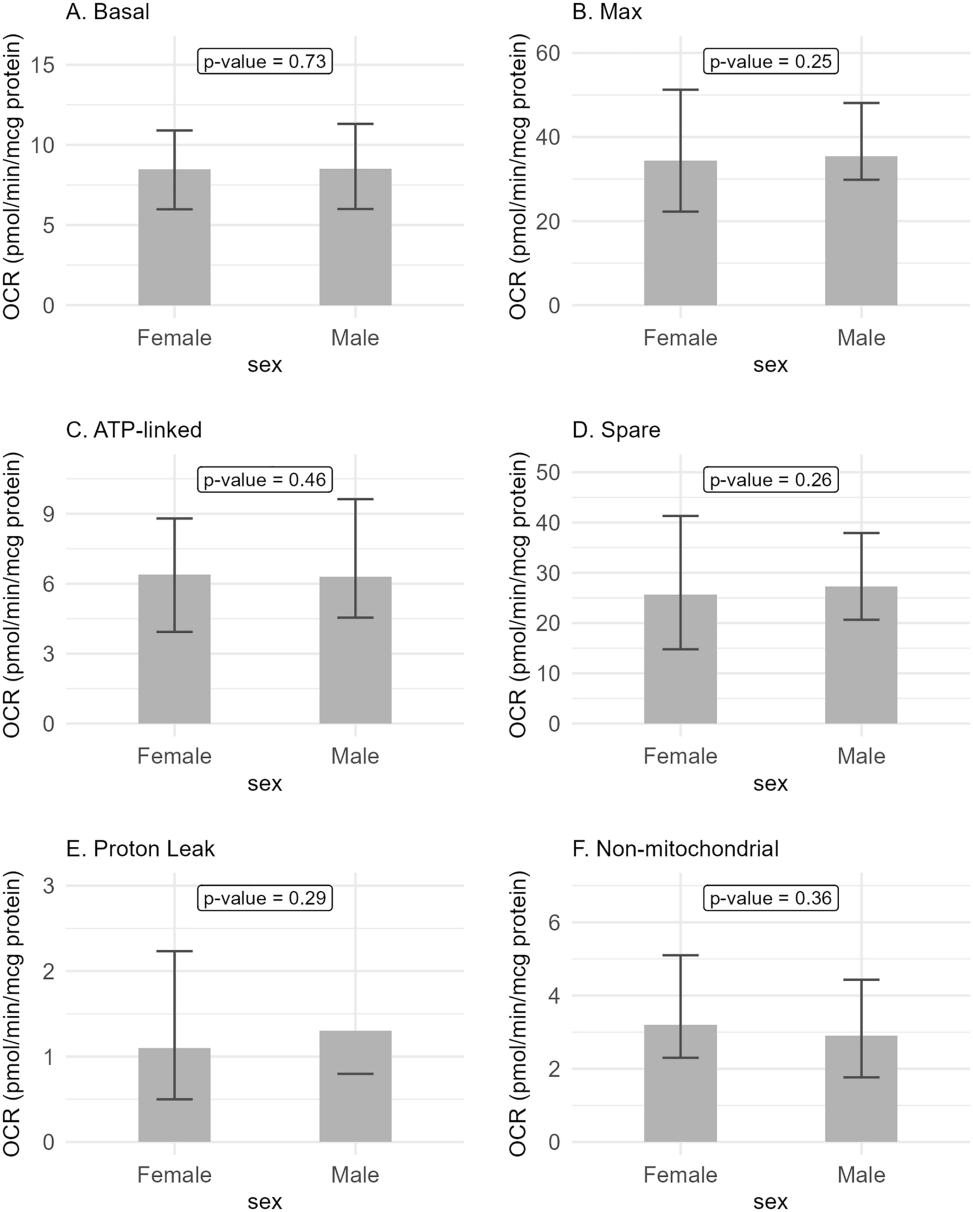
Oxygen consumption rate parameters and sex. There was no association between sex and OCR parameters.

**Fig 2:**
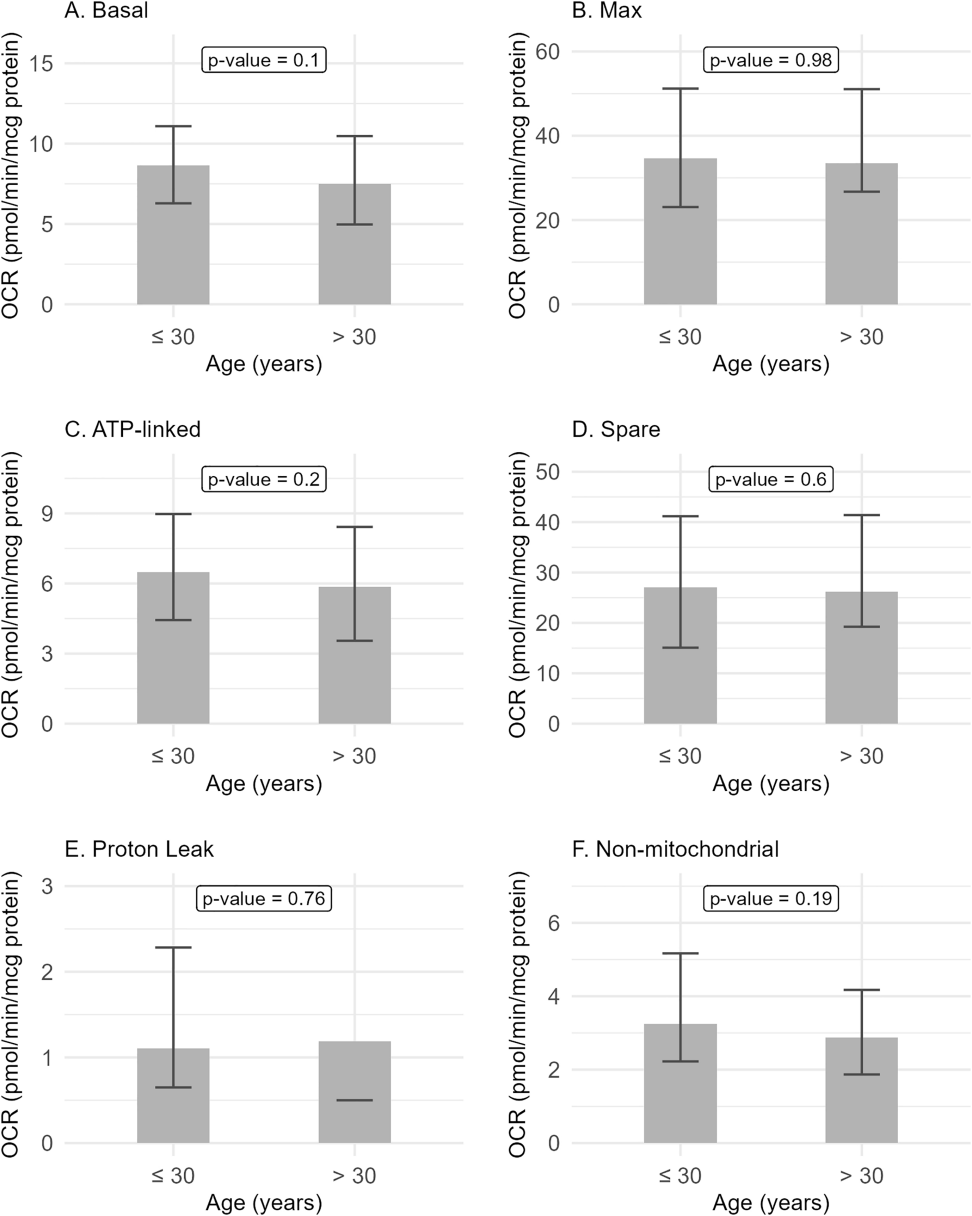
Oxygen consumption rate (OCR) parameters and age groups. Participants were divided into two age groups (≤ 30 years vs. > 30 years) to assess for any age-related effect on mitochondrial respiration. There was no association between age groups and OCR parameters.

**Fig 3:**
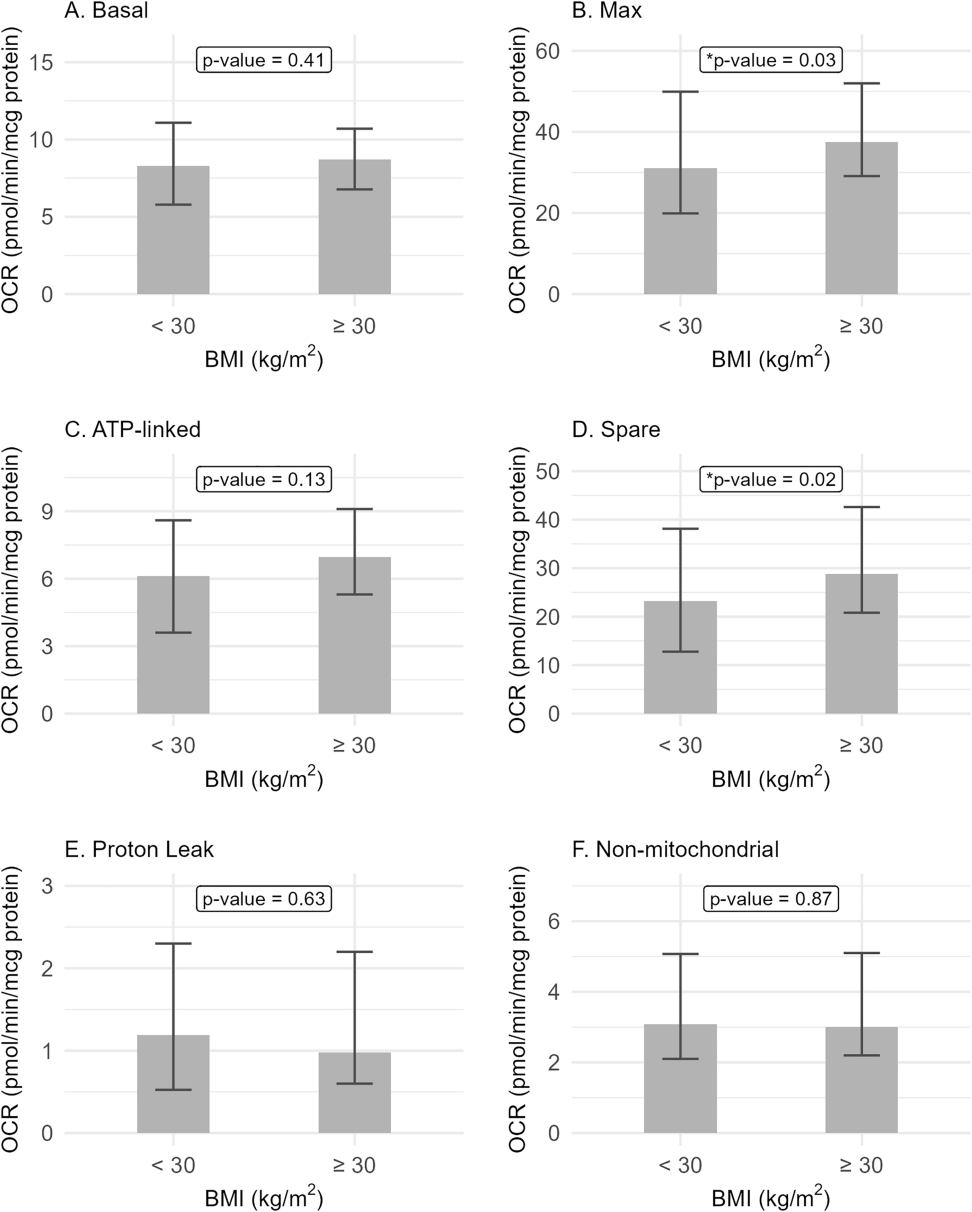
Oxygen consumption rate parameters and body mass index (BMI) groups. Maximal and spare respirations were associated with BMI groups. Other OCR parameters were not associated with BMI groups.

The OCR parameters were not associated with sex (Figs 1, S2 and Table 2). Controlling for age and BMI also did not reveal any significant association with sex (S3 Fig). The OCR parameters were also not associated with age as an ordinal variable with two levels (≤ 30 and > 30 years) (Figs 2, S4 and Table 3). Controlling for sex and BMI also did not reveal any significant association with age (S5 Fig). There was also no significant association with age as continuous variable (S6 Fig). Maximal and spare respirations were associated with BMI as an ordinal variable (p = 0.03 and p = 0.02, respectively) (Figs 3, S7 and Table 4). Similar findings were seen even when controlled for age and sex (S8 Fig). No association was seen with BMI as continuous variable (S9 Fig). De-identified data, including demographics and OCR parameters, can be found in the supporting file (S1 File).

**Table 2:** Mitochondrial respiration and sex.

OCR	Sex	Median (IQR)	p-value
Basal	Female	8.5 (6, 10.9)	0.73
	Male	8.5 (6, 11.3)	
Maximal	Female	34.4 (22.2, 51.3)	0.25
	Male	35.4 (29.8, 48.1)	
ATP-linked	Female	6.4 (3.9, 8.8)	0.46
	Male	6.3 (4.5, 9.6)	
Spare	Female	25.7 (14.8, 41.3)	0.26
	Male	27.3 (20.6, 37.9)	
Proton leak	Female	1.1 (0.5, 2.2)	0.29
	Male	1.3 (0.8, 3)	
Non-mito	Female	3.2 (2.3, 5.1)	0.36
	Male	2.9 (1.8, 4.4)	

*n* = 145 for female and *n* = 39 for male; OCR, oxygen consumption rate; IQR, interquartile range; ATP, adenosine triphosphate; Non-mito, non-mitochondrial.

**Table 3:** Mitochondrial respiration and age.

OCR	Age group	Median (IQR)	p-value (trend)^‡^	p-value (pairwise)^§^
Basal	All^†^	Effect: −0.01 (−0.14, 0.11)	0.82	–
	≤ 30	8.6 (6.3, 11.1)		0.10
	> 30	7.5 (5, 10.5)		
Maximal	All	Effect: −0.11 (−0.48, 0.27)	0.57	–
	≤ 30	34.6 (23.1, 51.2)	–	0.98
	> 30	33.5 (26.7, 51.1)		
ATP-linked	All	Effect: 0.00 (−0.10, 0.09)	0.95	–
	≤ 30	6.5 (4.4, 9)	–	0.20
	> 30	5.8 (3.5, 8.4)		
Spare	All	Effect: −0.01 (−0.3, 0.27)	0.92	–
	≤ 30	27.1 (15.1, 41.2)	–	0.60
	> 30	26.2 (19.2, 41.4)		
Proton leak	All	Effect: 0.02 (0, 0.05)	0.07	–
	≤ 30	1.1 (0.7, 2.3)	–	0.76
	> 30	1.2 (0.5, 3.2)		
Non-mito	All	Effect: −0.02 (−0.06, 0.01)	0.19	–
	≤ 30	3.2 (2.2, 5.2)	–	0.19
	> 30	2.9 (1.9, 4.2)		

*n* = 138 for ≤ 30 years and *n* = 46 for > 30 years; ^†^Effect of age as continuous variable on OCR. ^‡^Age as a continuous variable. ^§^Age as an ordinal variable. OCR, oxygen consumption rate; IQR, interquartile range; ATP, adenosine triphosphate; Non-mito, non-mitochondrial.

**Table 4:** OCRs and BMI.

OCR	BMI (kg/m^2^)	Median (IQR)	p-value (trend)^‡^	p-value (pairwise)^§^
Basal	All^†^	Effect: 0.0 (−0.14, 0.14)	1.00	–
	≤ 30	8.3 (5.8, 11.1)	–	0.41
	> 30	8.7 (6.8, 10.7)		
Maximal	All	Effect: 0.57 (−0.18, 1.32)	0.14	–
	≤ 30	31.1 (19.9, 49.9)	–	0.03*
	> 30	37.5 (29.1, 52)		
ATP-linked	All	Effect: 0.03 (−0.09, 0.15)	0.60	–
	≤ 30	6.1 (3.6, 8.6)	–	0.13
	> 30	7.0 (5.3, 9.1)		
Spare	All	Effect: 0.49 (−0.07, 1.04)	0.09	–
	≤ 30	23.2 (12.8, 38.1)	–	0.02*
	> 30	28.8 (20.8, 42.6)		
Proton leak	All	Effect: −0.02 (−0.06, 0.01)	0.15	–
	≤ 30	1.2 (0.5, 2.3)	–	0.63
	> 30	1 (0.6, 2.2), n = 6		
Non-mito	All	Effect: 0.01 (−0.07, 0.09)	0.8	–
	≤ 30	3.1 (2.1, 5.1)	–	0.87
	> 30	3.0 (2.2, 5.1)		

*n* = 106 for ≤ 30 kg/m^2^ and *n* = 65 for > 30 kg/m^2^; ^†^Effect of body mass index (BMI) as continuous variable on OCR. ^‡^BMI as a continuous variable. ^§^BMI as an ordinal variable. OCR, oxygen consumption rate; IQR, interquartile range; ATP, adenosine triphosphate; Non-mito, non-mitochondrial.

## Discussion

This study investigated mitochondrial respiration in PBMCs of a large number of healthy adult participants and to contribute to the existing body of literature on baseline mitochondrial bioenergetics dataset. We assessed the association between mitochondrial respiration and various demographic and anthropometric parameters to better understand the factors that affect mitochondrial respiration. This study is unique in that it exclusively examined healthy individuals without any previously diagnosed medical conditions and included a substantially larger sample size than prior studies. We found no significant associations between OCR parameters and sex and age, but found significant association between maximal and spare respiration with BMI.

The potential influence of sex on mitochondrial respiration has been of considerable interest, with studies producing somewhat conflicting results. Early work by Silaidos et al., involving 15 female and 9 male participants, reported that women exhibited higher mitochondrial content (as measured by citrate synthase activity), greater uncoupled respiration and respiratory capacity, and higher intracellular ATP levels compared with men [41]. However, larger analyses have generally not supported a consistent sex difference. A comprehensive meta-analysis by Junker et al., including 2,258 participants across 50 studies, found no significant overall differences in mitochondrial respiration between sexes. Specifically in PBMCs, they noted that female had higher respiration when normalized to citrate synthase activity, whereas male had higher respiration when normalized to cell count. They also found that female have higher mitochondrial content than male [47]. Similarly, Ehinger et al. found no significant sex differences in PBMC respiration [33]. Our study showed overall consistent results, showing no significant differences in any respiration parameters between sexes even after adjusting for age and BMI. Taken together, these findings suggest that while mitochondrial content may differ between sexes, overall mitochondrial function appears broadly similar in female and male.

The effect of age on mitochondrial function has long been of interest, as mitochondrial respiration has been thought to decline gradually with aging, potentially due to the accumulation of mutations in mitochondrial DNA (mtDNA) [48,49]. While some studies in human skeletal muscle have shown age-related reductions in mitochondrial respiration [50–53], findings in PBMCs have been mixed. An early study by Alonso et al. demonstrated a significant decline in basal, maximal, and spare respiration in PBMCs of patients with mitochondrial disease compared with healthy controls, even after normalization to mtDNA content, though the study was limited by a small number of healthy controls (n = 38) [38]. In contrast, Tessema et al. examined 518 individuals (493 patients and 25 healthy controls) and found that PBMC maximal and spare respiration increased with age, particularly in older men [39]. Conversely, a recent large population study in Thailand by Sriwichaiin et al. involving 619 middle-aged (53–64 years) and older (>65 years) participants reported that all respiratory parameters were lower in older adults, even after adjusting for sex and medical history [54]. Similarly, Liabeuf et al. observed that PBMC respiration declined with increasing frailty, particularly in frail men [55]. However, more recently, Ehinger et al., in one of the largest studies to date (308 participants, including 91 healthy controls), found age-related changes in PBMC respiration to be small or absent and reported no significant difference in citrate synthase activity, a marker of mitochondrial content, across age groups [33]. This study, which controlled for sex and comorbidities, provided strong evidence that age may have a limited effect on PBMC mitochondrial respiration. In our study, which included the largest number of healthy participants to date, we also observed no statistically significant associations with age even after controlling for sex and BMI. There were trends toward lower basal, maximal, ATP-linked, and spare respiration, and higher proton leak with increasing age although these changes were small in magnitude. These findings may have been influenced by the limited number of participants over 40 years of age, which reduced the statistical power to detect age-related effects.

Our findings of an association between maximal and spare respiration and BMI in PBMCs are overall consistent with several prior studies. Early work in skeletal muscle showed that mitochondrial respiration decreased by ~25% after weight loss [56] while citrate synthase activity remained stable demonstrating the positive association with BMI. More recent pediatric studies found that maximal and spare respiration in PBMCs correlated positively with BMI [57]. Another pediatric study showed that the OCR/ECAR ratio in PBMCs increased with BMI, suggesting a shift toward increased respiration likely to meet the elevated energy demands of obesity [58]. The association between mitochondrial respiration and BMI, especially for maximal and spare respiration, is likely multifactorial. Conceptually, maximal and spare respiration represent the mitochondrial “reserve” available under stress, enabling cells to respond to acute energetic challenges [59]. In the setting of obesity, PBMCs may recruit this reserve to respond to the combined effects of increased demand and systemic inflammation associated with obesity. For example, Bohm et al. showed that among obese patients, those with metabolically unhealthy obesity had higher PBMC respiration compared with metabolically healthy individuals, findings consistent with the thought that there is compensatory increase in mitochondrial activity to cope with greater metabolic demand and inflammation associated with metabolic syndrome [60]. In fact, multiple studies showed positive correlation between mitochondrial respiration and inflammatory markers. Shirakawa et al. showed that respiration in PBMCs were increased in obese insulin resistant patients and correlated with proinflammatory marker IL-6 [61]. Gebhardt also showed maximal respiration was positively associated with IL-8 while basal respiration was negatively correlated with TNF-α [62].

However, the association with BMI likely depends on the cell type. Previous studies examining different cell types have shown conflicting results. For example, Bohm et al. [60] demonstrated a positive correlation between mitochondrial respiration and BMI in adipocytes, and even within adipose tissue, visceral fat appeared more bioenergetically active than subcutaneous fat [63]. In contrast, studies in skeletal muscle, such as that by Fisher-Wellman et al. [64] reported no significant difference between insulin-sensitive lean and insulin-resistant obese individuals. The findings observed in PBMCs may reflect the fact that circulating immune cells are more responsive to systemic inflammation and metabolic stress than skeletal muscle tissue. Therefore, the findings of increased maximal and spare respiration in PBMCs with higher BMI in our study are likely driven by multiple contributing factors. Our cohort consisted of a healthy population without prior medical diagnoses; however, participants with higher BMI may still experience increased metabolic demand and low-grade systemic inflammation associated with obesity.

Normalization in mitochondrial respiration assays remains an important and unresolved issue, which no universally accepted standard to date. In our study, we seeded equal number of cells per well and normalized OCR measurements to total cellular protein content. While the use of internal normalization through pre-assay cell count has been the most common technique used for Seahorse assays [65], several studies have employed post-assay normalization, including total protein content [66–69], as done in our work, or genomic DNA content [36]. Our review of recent studies using Seahorse assays with PBMCs showed that majority of work utilized only the pre-assay cell count method for normalization [32,34,35,58,70–72]. Since the initiation of our study, additional methods such as post-assay cell count or DNA content fluorescent imaging have been introduced. These methods were not implemented in this work to maintain consistency in protocol over time. However, the lack of consensus on normalization approaches highlights an important area for methodological standardization, which is essential to facilitate reliable comparison and reproducibility across studies.

Lastly, it is important to consider the methodological differences in mitochondrial respiration measurement when interpreting our findings in the context of existing literature. The two most commonly used instruments for mitochondrial respirometry are the Seahorse XF Analyzer, a microplate-based phosphorescence system, and the Oroboros Oxygraph-2k (O2k), a chamber-based Clark-type amperometric system [73,74]. Microplate-based respirometry systems such as Seahorse have been increasingly adopted in recent years due to their high throughput and ease of use [73]. Comparative studies between the two platforms have demonstrated overall concordance in respiratory parameters, although absolute oxygen consumption rates may differ. For example, in fibroblasts, maximal respiration (electron transfer capacity) measured with Oroboros was higher than that obtained with Seahorse [75], whereas in platelets, Seahorse measurements yielded higher values [76]. For our study, which aimed to assess mitochondrial respiration in PBMCs from a large number of participants, the Seahorse system offered distinct advantages, including minimal sample volume requirements and the ability to process multiple samples in parallel (96-well format) with high throughput.

There are several important limitations of this study. First, this study evaluated OCRs in PBMCs only and the findings may not be generalizable to other cell types in the body. Second, due to the setting where the participants were recruited, the demographic and anthropometric parameters of the study participants were not balanced. This study had more females than males, predominantly young (≤ 30 years), mostly White, and non-Hispanic. This limits the generalizability of the findings. Despite this important limitation, we believe that the data and findings from this study make an important contribution to the limited existing work on this area. Lastly, previous studies have seen different results with PBMC isolation techniques and measurement normalization methods.

## Conclusions

In this study, we measured mitochondrial respiration in PBMCs from healthy adults to assess mitochondrial function in the absence of disease and to examine its association with sex, age, and body mass index (BMI). This work is unique in that it evaluated a large cohort of healthy individuals without any previously diagnosed medical conditions. The findings of this study enhance our understanding of mitochondrial function in health and contribute to the growing body of knowledge on mitochondrial bioenergetics dataset, which provides an important foundation for future research in mitochondrial function in various chronic and critical illnesses.

## Supporting information

S1 Fig A sample Seahorse XF Cell Mito Stress Test profile. (TIF)

S2 Fig Relative oxygen consumption rate parameters and sex. Relative values are calculated in reference to basal respiration. A. is missing for ease of comparison to Fig 1.
(TIF)

S3 Fig Oxygen consumption rate parameters and sex. Parameters were controlled for age and BMI.
(TIF)

S4 Fig Relative oxygen consumption rate parameters and age groups. Relative values are calculated in reference to basal respiration. A. is missing for ease of comparison to Fig 2.
(TIF)

S5 Fig Oxygen consumption rate parameters and age groups. Parameters were controlled for sex and BMI.
(TIF)

S6 Fig Oxygen consumption rate parameters and age as a continuous variable. (TIF)

S7 Fig Relative oxygen consumption rate parameters and BMI groups. Relative values are calculated in reference to basal respiration. A. is missing for ease of comparison to Fig 3.
(TIF)

S8 Fig Oxygen consumption rate parameters and BMI. Parameters were controlled for age and sex.
(TIF)

S9 Fig Oxygen consumption rate parameters and BMI as a continuous variable. (TIF)

S1 File De-identified data. (CSV)
